# The Effect of Groove Shape on Molten Metal Flow Behaviour in Gas Metal Arc Welding

**DOI:** 10.3390/ma14237444

**Published:** 2021-12-04

**Authors:** Amin Ebrahimi, Aravind Babu, Chris R. Kleijn, Marcel J. M. Hermans, Ian M. Richardson

**Affiliations:** 1 Department of Materials Science and Engineering, Faculty of Mechanical, Maritime and Materials Engineering, Delft University of Technology, Mekelweg 2, 2628 CD Delft, The Netherlands; A.Babu@tudelft.nl (A.B.); M.J.M.Hermans@tudelft.nl (M.J.M.H.); I.M.Richardson@tudelft.nl (I.M.R.); 2 Department of Chemical Engineering, Faculty of Applied Sciences, Delft University of Technology, van der Maasweg 9, 2629 HZ Delft, The Netherlands; C.R.Kleijn@tudelft.nl

**Keywords:** gas metal arc welding (GMAW), melt-pool behaviour, joint shape design, computational modelling

## Abstract

One of the challenges for development, qualification and optimisation of arc welding processes lies in characterising the complex melt-pool behaviour which exhibits highly non-linear responses to variations of process parameters. The present work presents a computational model to describe the melt-pool behaviour in root-pass gas metal arc welding (GMAW). Three-dimensional numerical simulations have been performed using an enhanced physics-based computational model to unravel the effect of groove shape on complex unsteady heat and fluid flow in GMAW. The influence of surface deformations on the magnitude and distribution of the heat input and the forces applied to the molten material were taken into account. Utilising this model, the complex thermal and fluid flow fields in melt pools were visualised and described for different groove shapes. Additionally, experiments were performed to validate the numerical predictions and the robustness of the present computational model is demonstrated. The model can be used to explore the physical effects of governing fluid flow and melt-pool stability during gas metal arc root welding.

## 1. Introduction

Gas metal arc welding (GMAW) is a fusion-based joining technique that is widely employed in industry to join metallic parts and to produce high-integrity structures. The quality of the joints made using arc welding or the structures made using wire-arc additive manufacturing depend on chosen process parameters, material properties and boundary conditions [1,2,3]. Changes in operating variables can alter the magnitude and distribution of the heat input and forces applied to the molten material in melt pools (such as Marangoni, Lorentz, thermal buoyancy forces and arc plasma shear stresses and pressures), affecting fluid flow in the pool and in turn the properties, structure and quality of products [2]. Correct control of melt-pool behaviour during arc welding is crucial to produce joints with desired properties [4].

One of the challenges for development, qualification and optimisation of arc welding processes lies in characterising the complex melt-pool behaviour which exhibits highly non-linear responses to variations of process parameters [5]. Trial-and-error experiments are often employed to realise appropriate processing parameters to achieve the desired properties. Such an experimental approach is costly and time inefficient and a successful processing for a specific configuration (e.g., material system, welding machine and joint shape) might not apply to a different configuration. Moreover, experimental identification of the effects of various parameters on the melt-pool behaviour is generally complicated due to the high-temperature, rapid solid–liquid phase transformation, opacity and fast dynamics of the molten metal flow [4]. Simulation-based approaches offer an understanding of the melt-pool behaviour during welding and additive manufacturing and can serve as an alternative to experiments to explore the design space for process optimisation [1,6].

To date, focus has predominantly been placed on developing numerical simulations to describe melt-pool behaviour in arc welding of flat plates without a groove (i.e., bead-on-plate welding, see for instance, [7,8,9,10,11,12,13,14,15]); however little attention has been paid to understanding the effect of joint shape on complex heat and molten metal flow. Zhang et al. [16,17] developed a three-dimensional model in a body-fitted coordinate system to describe the effects of various driving forces on heat and fluid flow in the melt pool during GMAW fillet welding. Hu and Tsai [18] developed a comprehensive model to simulate unsteady molten metal flow and heat transfer in melt-pools during GMA welding of a thick plate with V-groove. These studies only focus on partially penetrated pools and do not report the effect of different joint shapes on molten metal flow behaviour. Chen et al. [19] studied the effect of the opening angle of a V-groove on melt-pool behaviour during relatively high-current GMAW (welding current I=340 A) using a computational model developed on the basis of a body-fitted coordinate system. They reported that changes in the opening angle have an insignificant effect on the flow pattern in the pool but can affect the velocity and temperature distribution and thus the pool shape. Using the Abel inversion method, Cho and Na [20] reconstructed the emissivity distribution of an arc plasma and argued that the application of V-grooves in arc welding can affect the arc plasma characteristics, changing the distribution of the power-density, arc pressure and electromagnetic forces [21]. On the basis of their previous studies [20,21], Cho et al. [22] employed an elliptically symmetric distribution functions for power-density and arc pressure (instead of an axisymmetric distribution) to simulate heat and fluid flow in GMAW of a plate with V-groove at different welding positions. Changes in the groove shape due to filler metal deposition and its effect on the distribution of power-density and arc-induced forces were not accounted for in previous models that are available in the literature. Further investigations are required to realise the influence of the joint shape on molten metal flow behaviour in GMAW, particularly for fully-penetrated melt pools.

Focusing on understanding the melt-pool behaviour during root-pass gas metal arc welding, with a particular interest in the effects of groove shape, a systematic numerical study was carried out in the present work. Three-dimensional calculations have been performed using a physics-based computational model to simulate the dynamics of heat and molten metal flow in GMAW. Additionally, experiments were performed to validate the numerical predictions. The present work explains the dynamics of internal molten metal flow in gas metal arc welding and provides an enhanced computational model for design space explorations.

## 2. Problem Description

**Figure 1:**
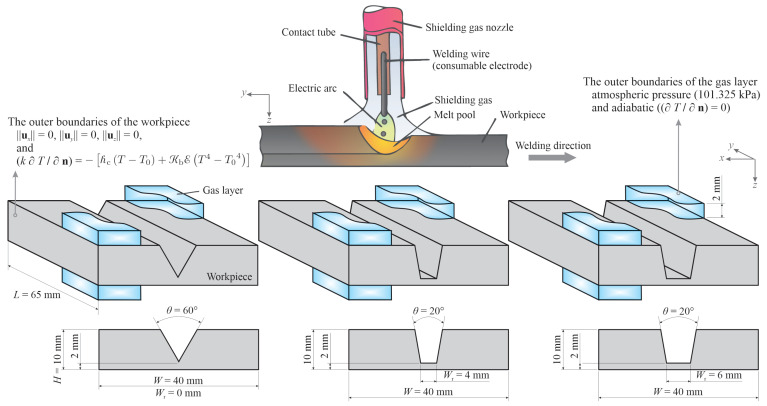
Schematic of gas metal arc welding and three different joint shapes studied in the present work. For the sake of clarity, only parts of the gas layer are shown. Here *W*_r_ refers to the width of flat region at the base of the groove, referred to as the root-leg.

In gas metal arc welding, an electric arc between a consumable electrode (filler metal) and a workpiece provides the thermal energy required for melting the material. Melting of the filler metal results in the periodic formation of molten metal droplets that successively impinge on the workpiece surface. Thermal energy input from the arc plasma as well as the thermal energy transported by the droplets leads to the formation of a melt pool that creates a joint after solidification (see Figure 1). In the present work, the effect of the groove shape on molten metal flow behaviour is studied for three different groove shapes, as shown schematically in Figure 1. A torch, which is perpendicular to the workpiece top-surface is adopted here and the contact-tip to workpiece distance (CTWD) is set to 18 mm. Different values of welding current ranging between 220 A and 280 A have been studied. Details of the welding parameters in the present work are listed in Table 1. The process parameters employed in the present work have been chosen based on preliminary trial experiments and are also comparable to those reported in previous independent studies on gas metal arc welding of steel plates with grooves (see, for instance, [17,18,21,22]). The plates are made of a stainless steel alloy (AISI 316L) and are initially at an ambient temperature of 300 K. The welding torch is initially located in the middle of the workpiece along the *x*-axis and 10 mm away from the leading-edge of the workpiece (i.e., *y* = 10 mm).

**Figure 2:**
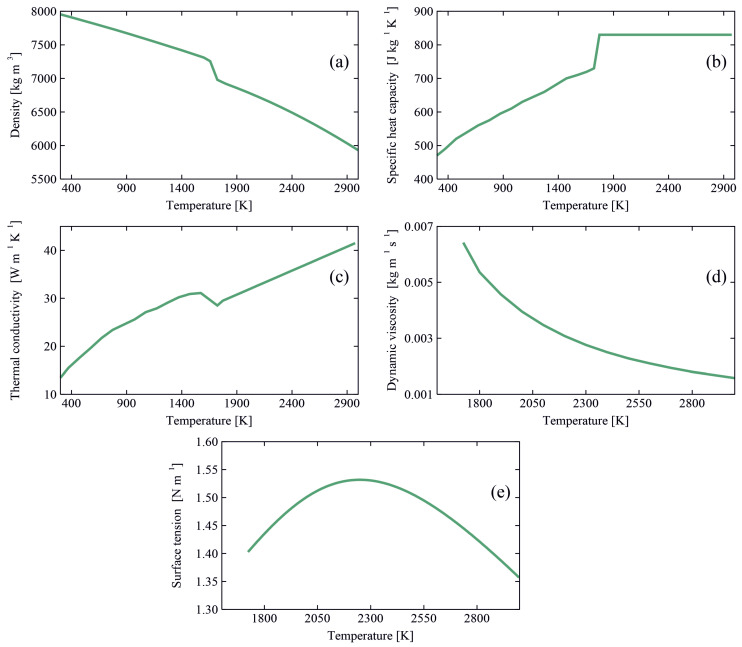
Temperature-dependent thermophysical properties of AISI 316L employed in the simulations. (**a**) density [26], (**b**) specific heat capacity [25], (**c**) thermal conductivity [25], (**d**) dynamic viscosity [26] and (**e**) surface tension [23].

The computational domain is defined in a stationary Cartesian coordinate system and is in the form of a rectangular cube that encompasses the metallic workpiece and two layers of gas above and below the workpiece. The incorporation of the gas layers allows tracking of surface deformations of the pool. To reduce the complexity of simulations and computation time, the melt-pool is decoupled from the arc plasma in the simulations. Accordingly, the heat input from the arc and the arc induced forces are defined as source terms for thermal energy and momentum. These source terms are adjusted dynamically during the calculations, as explained in Section 3, to account for the changes in the arc power and power-density distribution as well as the magnitude and distribution of the forces exerted by the arc plasma that occur due to melt-pool surface deformations and filler metal deposition. The conditions applied to the outer boundaries of the computational domain are shown in Figure 1. The outer boundaries of the plates are treated as no-slip walls, as the melt-pool does not reach them, and heat losses due to radiation and convection are accounted for. A fixed atmospheric pressure (101,325 Pa) is applied to the outer boundaries of the gas layers. The thermophysical properties of AISI 316L and the gas employed in the simulations are presented in Table 2 and Figure 2. The values for the surface tension are estimated using an empirical correlation proposed by Sahoo et al. [23], which takes the influence of surfactants (i.e., sulphur) into account. Employing a temperature-dependent density model, thermal buoyancy forces are accounted for in the simulations. In the present work, the properties of the shielding gas are assumed to be temperature-independent, which is a common assumption in numerical simulations of arc welding and additive manufacturing where the melt-pool is decoupled from the arc plasma [7,8,9,11,12,13,22]. This assumption is justifiable as the transport properties of the shielding gas (i.e., viscosity, density and thermal conductivity) are small compared to those of the molten metal, and thus changes in the shielding gas properties with temperature negligibly affect the numerical predictions of fluid flow in the melt pool [24].

## 3. Methods

### 3.1. Mathematical Model

A three-dimensional multiphase model has been developed to predict molten metal flow, heat transfer and associated surface movements in gas metal arc welding. In the present model, the fluids (i.e., the molten metal and the gas) are considered to be Newtonian and their densities are assumed to pressure-independent. Assuming that the fluid flows under consideration are in the continuum regime, the dynamics of heat and fluid flow in melt pools and their surroundings are governed by the equations of motion given by the conservation equations for mass, momentum and energy. Accordingly, the unsteady governing equations are cast as follows:

(1) $$\frac{D\rho }{Dt}=S_{m},$$

(2) $$\rho \frac{Du}{Dt}=\mu \nabla^{2}u-\nabla p+F_{d}+F_{s}+F_{b}+S_{m}\left(u_{s}-u\right),$$

(3) $$\rho \frac{Dh}{Dt}=\frac{k}{c_{p}}\nabla^{2}h-\rho \frac{D\left(\psi L_{f}\right)}{Dt}+S_{q}+S_{l}+S_{m}\left(L_{f}+\int_{T_{i}}^{T_{d}}c_{p}\;dT\right),$$

where, *ρ* is the density, *t* the time, **u** the relative fluid-velocity vector, **u**_s_ the fluid-velocity vector for the filler metal droplet, *p* the pressure, *μ* the dynamic viscosity, *c*_p_ the specific heat capacity, *k* the thermal conductivity, *T* the temperature, *h* the sensible heat, $\left(\psi L_{f}\right)$ the latent heat, and *S*_m_ the source term defined to model filler metal addition [27]. The subscripts ‘d’ and ‘i’ indicate the droplet and initial condition, respectively. The total enthalpy of the material H is the sum of the sensible heat *h* and the latent heat $\left(\psi L_{f}\right)$ and is defined as follows [28]:

(4) $$H=\left(h_{r}+\int_{T_{r}}^{T}c_{p}\;dT\right)+\psi L_{f},$$

where, $L_{f}$ is the latent heat of fusion, and ψ the local liquid volume-fraction. Here, the subscript ‘r’ indicates the reference condition. Assuming the liquid volume-fraction ψ to change linearly with temperature [28], its value can be calculated as follows:

(5) $$\psi =\frac{T-T_{s}}{T_{l}-T_{s}};\;T_{s}\leq T\leq T_{l},$$

where, $T_{s}$ and $T_{l}$ are the solidus and liquidus temperatures, respectively.

The volume-of-fluid (VOF) method [29] is adopted to capture the position of the gas–metal interface. In the VOF method, the scalar function ϕ indicates the local volume-fraction of a phase in a given computational cell and its value varies from 0 in the gas phase to 1 in the metal phase. Accordingly, computational cells with 0<ϕ<1 represent the gas–metal interface. The advection of the scalar function ϕ is described by the linear advection equation as follows:

(6) $$\frac{D\phi }{Dt}=\frac{S_{m}}{\rho }.$$

Accordingly, the effective thermophysical properties of the material in each computational cell are determined as follows:

(7) $$\xi =\phi \;\xi_{m}+\left(1-\phi \right)\xi_{g},$$

where, ξ corresponds to density ρ, viscosity μ, thermal conductivity *k* or specific heat capacity $c_{p}$, and subscripts ‘m’ and ‘g’ indicate metal or gas, respectively.

Solidification and melting occurs in the temperature range between $T_{l}$ and $T_{s}$ in the so-called ‘mushy zone’. To model the suppression of liquid velocities in solid regions, and damping of liquid velocities in the mushy zone, the sink term $F_{d}$ based on the enthalpy-porosity technique [30], is incorporated into the momentum equation and is defined as

(8) $$F_{d}=-C\;\frac{{\left(1-\psi \right)}^{2}}{\psi^{3}+\epsilon }\;u,$$

where, *C* is the mushy-zone constant and ϵ is a small constant, equal to $10^{-3}$, employed to avoid division by zero. Depending on the melting temperature range as well as the imposed boundary conditions, the value of the mushy-zone constant can affect the numerical predictions of solidification and melting simulations. The value of the mushy-zone constant should be assigned appropriately to avoid numerical artefacts in simulations of solid–liquid phase transformations, which is discussed in detail in [31]. In the present work, the value of the mushy-zone constant *C* was chosen to equal 10^7^ kg m^−2^ s^−2^ [31].

To model forces acting on the gas–metal interface such as surface tension, thermocapillary and arc plasma forces, the continuum surface force (CSF) model [32] is utilised. In the CSF model, surface forces are considered as volumetric forces acting on the material contained in grid cells in the interface region. The source term $F_{s}$ is included in Equation (2) as follows:

(9) $$F_{s}=f_{s}∥\nabla \phi ∥\frac{2\rho }{\rho_{m}+\rho_{g}}\;,$$

where, subscripts ‘g’ and ‘m’ indicate gas or metal, respectively. In Equation (9), $f_{s}$ is the surface force applied to a unit area, and the term $2\rho /\;\left(\rho_{m}+\rho_{g}\right)$ is employed to abate the effect of the large metal-to-gas density ratio by redistributing the volumetric surface-forces towards the heavier phase (i.e., the metal phase). In addition to surface forces, body forces (i.e., electromagnetic forces) are incorporated in the source term $F_{b}$ in Equation (2).

To model the thermal energy input to the material, the source $S_{q}$ is included in Equation (3). Moreover, heat losses from the workpiece surface due to radiation and convection are accounted for by including the sink term $S_{l}$ in Equation (3).

In gas metal arc welding, the surface force acting on the gas–metal interface $f_{s}$ includes an arc plasma term, capillary and thermocapillary forces, and is defined as follows:

(10) $$f_{s}=f_{a}+\gamma \kappa \hat{n}+\frac{d\gamma }{dT}\left[\nabla T-\hat{n}\left(\hat{n}\;\cdot \;\nabla T\right)\right],$$

where, γ is the surface tension, κ the surface curvature ($\kappa =\nabla \;\cdot \;\hat{n}$), $\hat{n}$ the surface unit normal vector ($\hat{n}=\nabla \phi /∥\nabla \phi ∥$), and $f_{a}$ the arc plasma force that comprises arc pressure $f_{p}$ and arc plasma shear stress $f_{\tau }$,

(11) $$f_{a}=f_{p}+f_{\tau }.$$

The arc pressure $f_{p}$ is determined as follows [33]:

(12) $$f_{p}=F_{p}\left[\frac{\mu_{0}I}{4\pi }\frac{I}{2\pi {\sigma_{p}}^{2}}\exp\left(\frac{-R^{2}}{2{\sigma_{p}}^{2}}\right)\right]\hat{n},$$

where, $\mu_{0}$ is the vacuum permeability equal to $4\pi \;\cdot \;10^{-7}\;H/m$, and *I* is the current. The distribution parameter $\sigma_{p}$ (in metres) was determined on the basis of the experimental data reported by Tsai and Eagar [34] as follows:

(13) $$\sigma_{p}=7.03\times 10^{-2}\ell^{0.823}+2.04\times 10^{-4}I^{0.376},$$

where, *I* is the current, and *ℓ* the local arc length. Surface deformations can cause the total arc force applied to the melt-pool surface ($\underset{\forall }{\;\int \int \int \;}∥f_{p}∥\;dV$) to differ from the expected arc force ($\mu_{0}I^{2}/4\pi$) due to changes in ∥∇ϕ∥ [35,36]. This numerical artefact is negated by incorporating $F_{p}$, defined as follows:

(14) $$F_{p}=j\;\frac{\mu_{0}I^{2}}{4\pi }\frac{1}{\underset{\forall }{\;\int \int \int \;}∥f_{p}∥\;dV}.$$

The dimensionless factor j is employed, as suggested by Lin and Eagar [33] and Liu et al. [37], to match the theoretically determined arc pressure with experimentally measured values, and is calculated as follows:

(15) $$j=3+8\times 10^{-3}I,$$

with *I* the welding current.

The arc plasma shear stress $f_{\tau }$, which acts at a tangent to the surface, is defined as follows [38]:

(16) $$f_{\tau }=\left[\tau_{\max}\;g_{\tau }\left(R,\sigma_{\tau }\right)\right]\hat{t},$$

where, the maximum arc shear stress $\tau_{\max}$ [39,40], the arc shear stress distribution function $g_{\tau }$ [41] and the surface unit tangent vector $\hat{t}$ [38] were defined as follows:

(17) $$\tau_{\max}=7\times 10^{-2}I^{1.5}\exp\left(\frac{-2.5\times 10^{4}\bar{\ell }}{I^{0.985}}\right),$$

(18) $$g_{\tau }\left(R,\sigma_{\tau }\right)=\sqrt{\frac{R}{\sigma_{\tau }}}\exp\left(\frac{-R^{2}}{{\sigma_{\tau }}^{2}}\right),$$

(19) $$\hat{t}=\frac{r-\hat{n}\left(\hat{n}\;\cdot \;r\right)}{∥r-\hat{n}\left(\hat{n}\;\cdot \;r\right)∥}.$$

Here, $\bar{\ell }$ is the mean arc length, *I* the current, R the radius in *x*-*y* plane (i.e., $R=\sqrt{x^{2}+y^{2}}$), and r the position vector in the *x*-*y* plane. The distribution parameter $\sigma_{\tau }$ (in meters) is assumed to be a function of the mean arc length $\bar{\ell }$ and current *I* and was approximated using the data reported by Lee and Na [39]:

(20) $$\sigma_{\tau }=1.387\times 10^{-3}+I^{-0.595}{\bar{\ell }}^{0.733}.$$

$F_{b}$ in Equation (2) is the body force, which comprises electromagnetic and gravity forces. The electromagnetic force was computed using the model proposed by Tsao and Wu [42] transformed into a body-fitted coordinate system. Hence, the body forces are defined as follows:

(21) $${f_{b}}_{x}=\frac{-\mu_{0}I^{2}}{4\pi^{2}{\sigma_{e}}^{2}R}\exp\left(\frac{-R^{2}}{2{\sigma_{e}}^{2}}\right)\left[1-\exp\left(\frac{-R^{2}}{2{\sigma_{e}}^{2}}\right)\right]{\left(1-\frac{z-z^{\prime }}{H_{m}-z^{\prime }}\right)}^{2}\left(\frac{x}{R}\right),$$

(22) $${f_{b}}_{y}=\frac{-\mu_{0}I^{2}}{4\pi^{2}{\sigma_{e}}^{2}R}\exp\left(\frac{-R^{2}}{2{\sigma_{e}}^{2}}\right)\left[1-\exp\left(\frac{-R^{2}}{2{\sigma_{e}}^{2}}\right)\right]{\left(1-\frac{z-z^{\prime }}{H_{m}-z^{\prime }}\right)}^{2}\left(\frac{y}{R}\right),$$

(23) $${f_{b}}_{z}=\frac{-\mu_{0}I^{2}}{4\pi^{2}R^{2}H_{m}}{\left[1-\exp\left(\frac{-R^{2}}{2{\sigma_{e}}^{2}}\right)\right]}^{2}\left(1-\frac{z-z^{\prime }}{H_{m}-z^{\prime }}\right)+\rho g.$$

Here, $z^{\prime }$ is the position of the melt-pool surface in *x*-*y* plane at a given time *t*, g the gravitational acceleration vector, and the distribution parameter for the electromagnetic force $\sigma_{e}$ is the same as $\sigma_{p}$, according to Tsai and Eagar [34]. It should be noted that the current-density profile is assumed to be Gaussian in the model proposed by Tsao and Wu [42] to compute the electromagnetic forces. Further studies are required to develop a generic model to approximate the evolution of the current-density profile during gas metal arc welding [43,44,45].

The thermal energy provided by the arc is modelled by adding the source term $S_{q}$ to the energy Equation (3) and was defined as

(24) $$S_{q}=F_{q}\left[\frac{\eta_{a}IU}{2\pi {\sigma_{q}}^{2}}\exp\left(\frac{-R^{2}}{2{\sigma_{q}}^{2}}\right)∥\nabla \phi ∥\frac{2\;\rho \;c_{p}}{{\left(\rho \;c_{p}\right)}_{m}+{\left(\rho \;c_{p}\right)}_{g}}\right],$$

where, the arc efficiency $\eta_{a}$ is defined as follows:

(25) $$\eta_{a}=\eta_{p}-\eta_{d}.$$

Here, $\eta_{p}$ is the process efficiency and is assumed to vary linearly with welding current from 77% at 200 A to 72% at 300 A [46], and $\eta_{d}$ is the efficiency of thermal energy transfer by molten metal droplets, which is defined as follows:

(26) $$\eta_{d}=\frac{q_{d}}{IU}\;,$$

with $q_{d}$ the thermal energy content of the droplets that are assumed to be spherical. $q_{d}$ is defined as follows:

(27) $$q_{d}=\rho_{d}\frac{4}{3}\pi r_{d}^{3}\left(L_{f}+\int_{T_{i}}^{T_{d}}c_{p}\;dT\right)f_{d},$$

where, $r_{d}$ is the radius of molten metal droplet. The droplet temperature $T_{d}$ was approximated to 2500 K, based on the experimental data reported by Soderstrom et al. [47]. $f_{d}$ in Equation (27) is the frequency of droplet detachment, and is defined as:

(28) $$f_{d}=\frac{3u_{w}r_{w}^{2}}{4r_{d}^{3}},$$

where, $r_{w}$ is the radius of the welding wire, and $u_{w}$ the wire feed rate. For metal transfer in the spray mode, the radius of the molten metal droplets and the welding wire are assumed to be the same. Accordingly, the magnitude of molten metal droplet velocity $u_{d}$ just after detachment was approximated using the correlation proposed by Lin et al. [48]:

(29) $$u_{d}=\frac{I}{2\pi r_{d}}\sqrt{\frac{3\mu_{0}}{\rho_{d}}}G,$$

where, *I* is in Ampere, $r_{d}$ the radius of the droplet in meters, $\mu_{0}$ the vacuum permeability in Hm^−1^, $\rho_{d}$ the density of the molten droplet in kg m^−3^, and *G* a dimensionless constant introduced to obtain agreement with experimental measurements equal to 0.98 for steel electrodes.

The process voltage *U* was assumed to be a function of arc length *ℓ* and welding current *I* [49,50,51], and was determined as follows:

(30) $$U=U_{w}+U_{o}+U_{a}.$$

Here, $U_{w}$ is the wire voltage assumed to be constant and equal to 7 V [50], and $U_{o}$ the sum of the electrode fall voltages is assumed to be a function of welding current *I*:

(31) $$U_{o}=C_{I}I+10,$$

with $C_{I}$ the coefficient of variation of the electric fall voltage with current equal to 0.016 V A^−1^ [50,51]. $U_{a}$ in Equation (30) is the arc column voltage:

(32) $$U_{a}=C_{e}\ell ,$$

with $C_{e}$ the electric field strength equal to 1.09 V mm^−1^ [50,51]. On the basis of the data reported by Tsai and Eagar [34], the distribution parameter $\sigma_{q}$ (in meters) was determined as follows:

(33) $$\sigma_{q}=1.61\times 10^{-1}\ell^{0.976}+2.23\times 10^{-4}I^{0.395}.$$

The adjustment factor $F_{q}$ was employed to negate changes in the total heat input due to changes in surface morphology [52,53], which is defined as follows:

(34) $$F_{q}=\frac{\eta IU}{\underset{\forall }{\;\int \int \int \;}S_{q}\;dV}.$$

It should be noted that the source term $S_{q}$ is only applied to the top surface of the workpiece.

The sink term $S_{l}$ was added to the energy equation to account for heat losses due to radiation and convection, and is determined as follows:

(35) $$S_{l}=-\left[h_{c}\left(T-T_{0}\right)+K_{b}E\left(T^{4}-{T_{0}}^{4}\right)\right]∥\nabla \phi ∥\frac{2\;\rho \;c_{p}}{{\left(\rho \;c_{p}\right)}_{m}+{\left(\rho \;c_{p}\right)}_{g}}\;,$$

where, E is the radiation emissivity equal to 0.45 [54], $K_{b}$ the Stefan–Boltzmann constant, and $h_{c}$ is the heat transfer coefficient equal to 25 W m^−2^ K^−1^ [55].

### 3.2. Numerical Implementation

The computational model employed in the present work was developed within the framework of a proprietary finite-volume solver, ANSYS Fluent [56]. To implement the source terms in the governing equations and the surface tension model, user-defined subroutines programmed in the C programming language were used. The computational domain contains about 2.7 × 10^6^ non-uniform hexahedral cells, with the smallest cell spacing being set to 80 μm in the melt-pool region, which is sufficiently fine to obtain grid-independent solutions [35,36,52,53]. The cell spacing increases gradually from the melt-pool region towards the boundaries of the computational domain and the maximum cell size was limited to 400 μm. The central-differencing scheme with second-order accuracy was employed for spatial discretisation of momentum advection and diffusive fluxes. A first-order implicit scheme was employed for the time marching, and a fixed time-step size of 20 × 10^−5^ s was used to keep the value of the Courant number (Co=∥u∥Δt/Δx) below 0.25. To formulate the advection of the volume-fraction scalar field, an explicit compressive VOF method [57] was employed. Moreover, the PRESTO (pressure staggering option) scheme [58] and the PISO (pressure-implicit with splitting of operators) scheme [59] was employed for the pressure interpolation and coupling velocity and pressure fields, respectively. Simulations were executed in parallel on a high-performance computing cluster, each on 70 cores (AMD EPYC 7452) and the total run-time was about 290 h.

### 3.3. Experimental Setup and Procedure

**Figure 3:**
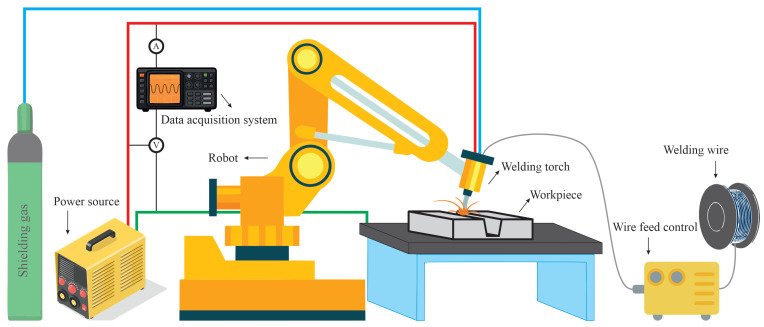
Schematic of the experimental setup utilised in the present work.

The general process parameters studied in the present work are introduced in Section 2. Figure 3 shows a schematic drawing of the experimental setup utilised in the present work. A Fronius CMT 5000i power source that was attached to a six-axis Fanuc robot was employed. Weld beads with a length of 80 mm were deposited on the workpiece with pre-machined grooves. Each experiment was repeated at least three times to ensure repeatability of the tests. The filler metal and the workpiece employed in the experiments were AISI 316L. Welding current and voltage were measured and recorded at a frequency of 5 kHz during the experiments using a Triton 4000 data acquisition system. Samples were cut after the experiments to extract transverse cross-sections. The cut samples were mounted and surface ground using silicon carbide (SiC) papers with grit sizes varying from 80 to 2000 grit. Finally, the samples were polished using colloidal alumina with particle sizes of 3 μm and 1 μm, respectively. Fusion zones were revealed by chemical etching with Kallings Reagent I (2 g CuCl_2_ + 40 mL HCl + 40 mL C_2_H_5_OH + 40 mL H_2_O) for 3 s. Macrographs of the fusion zones in the etched specimens were obtained using a Keyence digital microscope.

## 4. Results and Discussion

### 4.1. Model Validation

**Figure 4:**
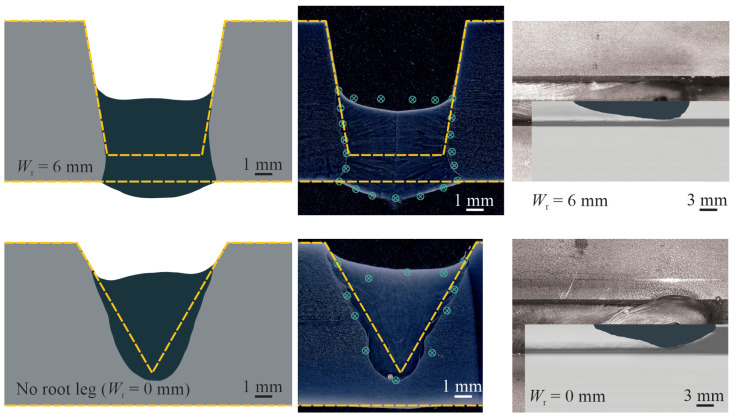
Comparison of the melt-pool shapes obtained from the present numerical simulations with experimental measurements for different groove shapes with a welding current of 280 A and a travel speed of 7.5 mm s^−1^. Regions shaded in dark grey show the melt-pool shape obtained from numerical simulations. The computational cells that, at any stage during the transient calculations of the melting and re-solidification process, contained molten metal were marked to visualise the melt-pool shape on the transversal cross-section. Green symbols on experimental data show the melt-pool boundary obtained from numerical simulations. Yellow dashed-lines indicate the joint shape before welding.

The reliability and accuracy of the present numerical predictions are benchmarked against experimentally measured melt-pool shapes. In this study, gas metal arc welding of workpieces with different groove shapes are considered, with a welding current of 280 A and a travel speed of 7.5 mm s^−1^. Figure 4 shows a comparison between the numerically predicted melt-pool shapes with those obtained from experiments for different groove shapes. The computational cells that, at any stage during the transient calculations of the melting and re-solidification process, contained molten metal were marked to visualise the melt-pool shape on the transversal cross-section. It is worth noting that the experiments were conducted after the numerical simulations, which means no calibration is performed to tune the numerical results. The results indicate a reasonable agreement between numerically predicted and experimentally measured melt-pool shapes. The maximum deviation between the predicted melt-pool dimensions and experimental measurements is found to be less than 10%, demonstrating the validity of the present numerical simulations. This deviation might be caused by uncertainties associated with the models employed to approximate the temperature-dependent material properties at elevated temperatures, the simplifying assumptions made to develop the computational model such as those employed to determine droplet size, velocity and temperature, and uncertainties in determining the boundary conditions in the model.

### 4.2. Thermal and Fluid Flow Fields

Once the arc ignites and the process begins, the welding wire heats up to the melting temperature and molten metal droplets form at the wire tip that detach periodically from the wire and deposit on the workpiece surface as shown schematically in Figure 1. The frequency of droplet detachment is directly proportional to the wire feed rate and ranges between 147 Hz and 187 Hz for the welding process parameters studied in the present work (see Table 1). To simplify the numerical simulations and as described in Section 3.1, the filler metal droplets are assumed to be spherical and are incorporated into the simulations with predefined velocity and temperature, which is a common practice in modelling melt-pool behaviour in gas metal arc welding (see, for instance, [7,8,18,21]). The qualitative melt-pool behaviour was found to be similar for different welding currents studied in the present work. Therefore, representative results for the cases with welding current of 220 A are shown and discussed in the paper.

**Figure 5:**
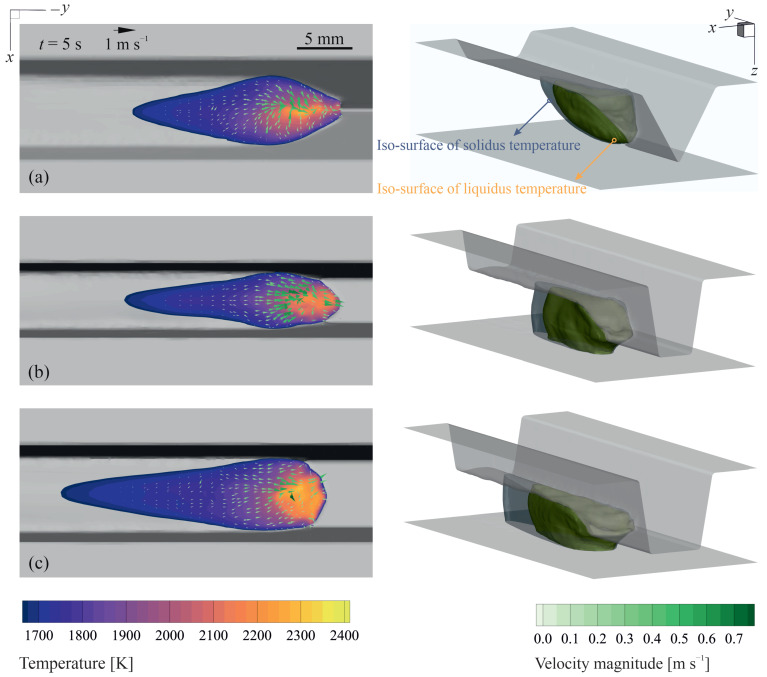
The numerically predicted thermal and fluid fields over the melt-pool surface (left column) and the corresponding pool shape (right column) for different joint shapes at *t* = 5 s. (**a**) groove angle $\theta =60^{\circ }$ and no root-leg ($W_{r}=0\;mm$), (**b**) $\theta =20^{\circ }$ and root-leg width $W_{r}=4\;mm$ and (**c**) $\theta =20^{\circ }$ and root-leg width $W_{r}=6\;mm$. Wire feed rate *u*_w_ = 7 m min^−1^, welding current *I* = 220 A, and travel speed *V* = 7.5 m s^−1^. The area between iso-surfaces of solidus and liquidus temperature shows the mushy region.

The thermal energy input from the plasma arc in addition to the thermal energy transported by the molten metal droplets result in the formation of a melt pool. For the process parameters studied in the present work (see Table 1), the melt pool grows over time and reaches a quasi-steady-state condition after about 3 s. Figure 5 shows a partial view of the workpiece encompassing the melt pool and the corresponding thermal and fluid flow fields over the melt-pool surface for different groove shapes at *t* = 5 s with wire feed rate u_w_ = 7 m min^−1^ and welding current *I* = 220 A. For the cases shown in Figure 5, the maximum surface temperature is less than 2310 K and the value of the temperature gradient of surface tension (∂γ/∂T) is mostly positive (see Figure 2e). Hence, the molten metal moves from the cold area close to the melt-pool rim towards the hot central region, primarily due to the Marangoni shear force induced over the surface. Molten metal streams from the melt-pool rim collide in the central region and form a complex unsteady asymmetric flow pattern in the pool. A similar flow pattern is observed experimentally in previous independent studies conducted by Wu et al. [60], Zhao et al. [61]. The maximum local molten metal velocity is about 0.7–0.8 m s^−1^ and corresponds to a Péclet number ($\mathrm{Pe}=\rho c_{p}D∥u∥/k$) larger than unity (O(400)), which signifies that advection dominates the energy transfer in the melt pool and that the process cannot be described adequately using a thermal model without considering fluid flow.

The results suggest that the energy transported to the surrounding solid material markedly affects the melt-pool shape. Although the total heat input to the material is the same for the cases shown in Figure 5, the melt-pool shapes differ notably for different groove shapes. It appears that increasing the width of the root-leg results in a decrease in the amount of heat diffused to the side walls of the groove as the height of the deposit layer reduces, leading to an increase in the length of the melt-pool as well as the mushy-zone (i.e., regions between the solidus and liquid iso-surfaces in Figure 5). Moreover, the average fluid velocity in the pool decreases with increasing width of the root-leg, which can be attributed to the decrease in the magnitude of temperature gradients generated over the surface. Among all the cases studied in the present work, full-penetration is observed only for those with root-leg, even for the case with welding current *I* = 280 A. Evidently, a higher welding current or a lower travel speed is required to achieve full penetration using grooves without root-leg (i.e., V-groove). However, increasing the welding current or reducing the travel speed results in an increase in total heat input to the material, which is often undesirable as it decreases the cooling rate and can adversely affect the properties of the joint, particularly when austenitic stainless steels are used [62,63,64]. Moreover, increasing the welding current can lead to a significant increase in arc force as the arc force is proportional to the welding current squared ($∥F_{\mathrm{arc}}∥\propto I^{2}$) [49], and thus limiting the welding current tolerance to avoid defects such as burn-through. Despite the fact that employing a root-leg can reduce the welding current required to achieve full penetration, employing a relatively wide root-leg may increase the number of welding passes required to fill the groove.

**Figure 6:**
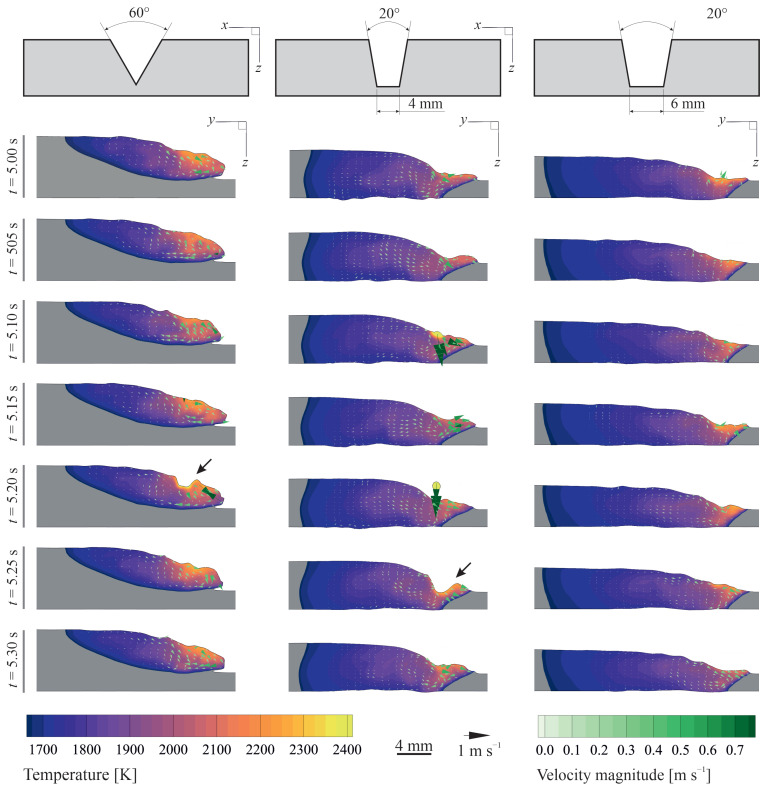
Melt-pool shape, temperature profile and velocity vectors in the x=0 plane for different joint shapes and time instances. (left column) groove angle $\theta =60^{\circ }$ and no root-leg ($W_{r}=0\;mm$), (middle column) $\theta =20^{\circ }$ and root-leg width $W_{r}=4\;mm$ and (right column) $\theta =20^{\circ }$ and root-leg width $W_{r}=6\;mm$. Wire feed rate *u*_w_ = 7 m min^−1^, welding current I=220 A, and travel speed *V* = 7.5 m s^−1^.

Figure 6 shows thermal and fluid flow fields in the x=0 plane for different joint shapes and time instances. The impingement of molten metal droplets on the surface disturbs the thermal and fluid flow field in the pool and results in the formation of a crater and a travelling wave over the melt-pool surface, as indicated by arrows in Figure 6. Moreover, the periodic molten metal droplet impingement on the melt pool enhances mixing in the melt pool. The molten metal droplet temperature ($T_{d}=2500\;K$) is above the critical temperature at which the sign of surface-tension temperature coefficient (∂γ/∂T) changes from positive to negative ($T_{\mathrm{cr}}\approx 2250\;K$); therefore, an outward fluid flow is induced on the surface in the region where the droplet is impinged due to Marangoni shear force. Soon after the droplet is merged with the melt pool, the crater closes due to surface tension and hydrostatic forces, and the surface temperature decreases to values less than 2310 K for which the value of ∂γ/∂T is mostly positive. The wave crests move radially outward towards the met-pool rim and are reflected by the solid edges of the pool. Interactions between the primary and reflected waves as well as the forces acting on the molten material result in complex melt-pool surface deformations and oscillations, as shown in Figure 5. For the cases studied in the present work, the frequency of the droplet transfer in relatively high (O(170 Hz)) and the droplet sizes are relatively small compared to the melt-pool dimension, resulting in a smooth weld bead with negligible ripple formation. Top-views of the melt-pool surface for different joint shapes and time instances are available in the supplementary materials.

## 5. Conclusions

Three-dimensional numerical simulations were performed to systematically investigate the effect of groove shape on melt-pool behaviour in root pass gas metal arc welding (GMAW). The effects of melt-pool surface deformations on power-density distribution and the forces applied to the molten material were accounted for in the present computational model. These effects are often neglected in numerical simulations of melt-pool behaviour in arc welding. Thermal and fluid flow fields in the melt pool are visualised and described for different groove shapes. Moreover, experiments were conducted to validate the numerical predictions.

Energy transfer in melt pools during gas metal arc welding is dominated by convection and thus thermal models without considering fluid flow cannot predict and describe the melt-pool shape with sufficient accuracy. The periodic impingement of molten metal droplets disturbs the thermal and fluid flow fields in the pool, resulting in an even more complex flow pattern. For the process parameters studied in the present work, full-penetration was observed only for the grooves with root-leg. Changes in the groove shape have an insignificant influence on the flow pattern over the surface, however, the groove shape affects the energy transfer to the surrounding solid material and thus alters the melt-pool shape and can affect the properties of the joint. The groove shape also affects the melt-pool oscillatory behaviour as it influences the reflection of the waves generated due to the molten metal droplet impingement. Moreover, the groove shape can affect the process window, which can be explored using the simulation-based approach described in the present work.

## Figures and Tables

**Table 1:** Welding parameters studied in the present work.

Parameter	Value	Unit
Welding current *I*	220–280	A
Arc voltage *U*	21.4–23.0	V
Wire feed rate *u*_w_	7.0–8.7	m min^−1^
Wire diameter *d*_w_	1.2 (0.045)	mm (inch)
Wire material	AISI 316L	–
Travel speed *V*	7.5	mm s^−1^
Shielding gas	97.5% Ar + 2.5% CO_2_	–
Shielding gas flow rate	20	l min^−1^
Inner diameter of the shielding cup	20	mm
CTWD	18	mm
Distance between the contact tip and the shielding cup edge	2	mm
Torch angle	90	∘

**Table 2:** Thermophysical properties of the stainless steel (AISI 316L) and the gas employed in the numerical simulations. Values for AISI 316 are taken from [25].

Property	Stainless Steel (AISI 316)	Gas	Unit
Density *ρ*	see Figure 2	1.623	kg m^−3^
Specific heat capacity *c*_p_	see Figure 2	520.64	J kg^−1^ K^−1^
Thermal conductivity *k*	see Figure 2	1.58 × 10^−2^	W m^−1^ K^−1^
Viscosity *μ*	see Figure 2	2.12 × 10^−5^	kg m^−1^ s^−1^
Latent heat of fusion ℒ	2.7 × 10^5^	–	J kg_−1_
Liquidus temperature *T*_l_	1723	–	K
Solidus temperature *T*_s_	1658	–	K

## Data Availability

The data generated in this study are available on reasonable request from the corresponding author.
